# Amylin in the Periphery

**DOI:** 10.1100/tsw.2003.17

**Published:** 2003-03-24

**Authors:** Peter J. Wookey, Loredanna Xuereb, Christos Tikellis, Mark E. Cooper

**Affiliations:** Department of Medicine, University of Melbourne, Austin and Repatriation Medical Centre, Repatriation Campus, Heidelberg West, Victoria, Australia

**Keywords:** amylin, islet amyloid polypeptide, hormone, growth factor, behavioral modifier, pancreatic islets, amyloid deposition, β-cell dysfunction, diabetes

## Abstract

Amylin (islet amyloid polypeptide) is a peptide synthesized principally in the b-cells of the pancreatic islets together with insulin and has actions as a hormone, growth factor, and modifier of behavior. As a hormone, amylin acts to modify gastric motility, renal resorption, and has metabolic actions. It is postulated that the principal function of amylin as a hormone is the activation of physiological processes associated with feeding. As a growth factor, amylin acts on bone cells, renal proximal tubular cells, and islet b-cells. Amylin has important targets in the brain that mediate its actions in the modification of behavior, including thirst and satiety. In man, amylin can form islet amyloid deposits, an event linked to the reduction of b-cell mass and loss of signal-secretion coupling. Recent evidence has defined a new role for monomeric amylin as a growth factor and regulator of b-cell mass that is postulated to be a key factor in pathophysiological processes that result in overt diabetes.

## INTRODUCTION

This mini-review is based on contemporary research that investigates the physiological roles of amylin and the pathophysiological consequences of variations in expression. Readers are directed to previous reviews for further discussion of prior research[1,2,3,4,5,6].

The contemporary view of many peptides present in the periphery includes the ability to elicit acute responses in target cells as hormones, to act as growth factors in particular contexts, and to alter behavior through actions in the brain. Amylin, discovered about 15 years ago[7,8], displays all these characteristics. The key actions of amylin as a hormone involved in gut motility, putative renal resorption, and metabolic outcomes, and as a peptide regulating thirst and satiety, provide the rationale that one of amylin's primary physiological roles is the activation of systems associated with feeding[4,9].

There are some peculiar features of amylin that have suggested a crucial role in the etiology and development of diabetes and associated complications. These features include the major site of biosynthesis being in the β-cells of the pancreatic islets[8], which is also the principal site for the biosynthesis of insulin. Amylin from humans[7,8] and other animals, including cats[8,10], has the potential to form amyloid deposits in the islets, thereby contributing to β-cell destruction, resulting in overt diabetes[11]. Indeed, the description of amyloid in the pancreas in association with diabetes is 100 years old, although its composition was only delineated 15 years ago[7,8]. The potential role of monomeric amylin as a growth factor is also explored in this article, as a mechanism influencing β–cell mass.

One of the major obstacles in the delineation of the involvement of amylin and amyloid deposition in these pathophysiological phenomena, and in the further exploration of amylin biology in general, is the paucity of nonpeptide antagonists and agonists, which one would predict would be forthcoming with increased knowledge of the primary amylin receptor(s).

## SITES OF AMYLIN BIOSYNTHESIS

The major site of amylin biosynthesis lies in the pancreatic islet β-cells, but secondary sites are found in the gastrointestinal tract[12,13], dorsal root ganglia[14,15,16], and in the developing kidney[18], where expression appears tightly controlled (discussed below[18]). In diabetic states little is known about expression from these secondary sites and any significance that this might have for the etiology of the disease and associated complications.

## HUMAN AMYLIN, NATURAL GENE MUTATIONS, AND DIABETES

The composition of amyloid deposits and how this influences β-cell function are among the key issues in the understanding of the pathogenesis of noninsulin-dependent diabetes melitus (NIDDM), a disorder for which there is increasing awareness of the role of β-cell dysfunction and atrophy[11]. Related to these issues is the mechanism(s) of amyloidogenesis, which in some cases is deleterious and in others apparently better tolerated by islet cells. This may be reflected in the fact that although 57%[19] to >90%[20] of NIDDM subjects display amyloid deposits in the islets, many nondiabetic individuals also show pancreatic amyloid deposition[8,19].

One of the early events in the pathogenesis of diabetes is considered to be an alteration in the signal-secretion coupling that finally results in the constitutive secretion of β-cell hormones and prohormones[21]. Furthermore, amylin has been found to modulate and/or inhibit insulin secretion in human studies[22]. A further question in relation to these putative changes in β-cell responses might involve the efficacy of amylin as a growth factor for β-cells (discussed below) and any role that reduced β-cell mass might have in the events leading to overt diabetes.

Investigations using biophysical techniques have identified sequences of human proamylin that may contribute to the processes of amyloid formation including phenylalanine-23[23] and sequences in the amino and carboxy terminal regions[24,25,26]. A molecular-recognition and self-assembly domain of IAPP/amylin has been identified in recent experiments[27]. Deamination of asparagines in this region dramatically affects aggregation of amyloidogenic peptides[28]. The finding that 2.6% of the Japanese[29,30] NIDDM population carried a mutation that resulted in a serine-20-> glycine conversion, compared to 0.8% of the total population (also in Hong Kong[31]) suggests that specific amino acids within the amylin molecule play a role in the pathogenesis of NIDDM[32]. A heparin-binding domain that may play a role in amyloidogenesis has also recently been described[33]. The prohormone convertase processing site is also located in this domain[34]. Such observations may ultimately lead to the identification of new factors that influence amyloidogenesis and are useful in the treatment of type II diabetes.

## TRANSGENIC MOUSE MODELS THAT EXPRESS HUMAN AMYLIN

A useful experimental model to study the amyloidogenic effects of human amylin and variants is the transgenic mouse that expresses human amylin. Eight research groups have reported the results of studies using this model[35,36,37,38,39,40,41,42]. One general theme, i.e., that the amount of secretion of human amylin determines the level of amyloidosis, was reiterated in a recent paper in which amounts of dietary fat[43] induced islet amyloidosis and impaired insulin secretion. These models are also potentially important to test candidate drugs effective in the inhibition and reversal of amyloidosis[44]. These studies varied in their conclusions but it is clear that there are still unknown factors important in the progression to overt diabetes following deposition of amyloid proteins. It is likely that the toxicity of particular amyloid deposits is dependent also on the state of the β-cells, and that human β-cells may behave differently from their mouse counterparts. Furthermore, the identification of some upstream mutations[45], which are linked to a propensity to develop diabetes, may corroborate the general view that the amount of amylin secreted is also a contributing factor. Some important upstream elements include Pax-4 regulatory elements that mediate expression of amylin and insulin[46]. Pax-4–deficient mice do not develop β- and ∂-cells, and die shortly after birth from diabetes**.**

## AMYLIN AS A HORMONE

Early studies highlighted the hyperglycaemic effects of amylin compared to glucagon[47], although the physiological mechanisms have not been clarified. Over a longer period of time, rats infused into the third ventricle with an antagonist of amylin ate more food and accumulated body fat without significant changes in body weight[48]. Thus the amylin concentration of the cerebrospinal fluid may contribute to long-term energy balance. Such a mechanism may also play a role in the glucagonostatic effect of amylin that is thought to be extrapancreatic in origin[49]. A 4-h infusion of amylin also significantly altered liver and muscle triglycerides without changes to steady-state serum levels. It is not clear what is the pathway of action and whether it is local or mediated through the CNS as discussed above[50]. Furthermore and in contrast to the results of antagonist infusion in the ventricle, subcutaneous administration of amylin in mice over a longer period increased bone mass and fat[51]. The possible role of amylin in lipid metabolism has been further explored in metabolic models infused with an amylin peptide antagonist[52].

In the periphery, amylin has important functions in proximal tubules of the kidney where it stimulates sodium/water resorption[53]. To explain its renal actions, high-affinity amylin binding sites have been identified on the proximal tubules[54]. Furthermore, intracellular, amylin immunoreactive vesicles have been detected within these tubules (data not shown).

The actions of amylin in the renal cortex are thought to include the maintenance of the polarity of proximal epithelial cells[4]. These functions, together with sustained activation of amylin receptors[55], are considered important in the pathogenesis of hypertension[56] and may be linked to renal complications associated with diabetes.

## AMYLIN IN THE MODIFICATION OF BEHAVIOR

Although this brief review concentrates on “amylin in the periphery”, some research relevant to its actions in the CNS and modifications of behavior are important to discuss for a more complete picture of the overall physiological role(s), particularly related to feeding.

The known actions of amylin as a hormone capable of behavioral modifications are equally diverse by increasing satiety[9,57], thirst[58], memory[59,60], and motor activity by interfering with central dopaminergic transmission[61]. Furthermore, effects via the PNS have been reported including inhibition of gastric emptying[13,62,63] and reduced nociception in the amylin gene-deletion mouse[64].

Two main regions of the circumventricular organs in the brain mediate peripheral variations in amylin levels. These include effects on drinking and feeding behavior via receptors in the *subfornical organ*[58,65,66] and *area postrema*[67,68], respectively.

The pathways of enhanced neural activity following amylin activation in the CNS are not yet defined but are likely to involve the histaminergic system[69], in particular H1 receptors. Evidence has also been presented implicating part of the dopaminergic system[70].

The *nucleus accumbens,* in which high-affinity amylin binding sites have been found in high density[71,72], may be one internal region processing these peripheral signals[73]. Furthermore, neuropeptide Y–induced feeding at the level of the hypothalamus is markedly attenuated by infusion of amylin[74]. The definition of the CNS pathways at the level of the neuron may be possible by identifying c-Fos activation, cGMP formation[67] and electrophysiological studies.

## AMYLIN AS A GROWTH FACTOR

Amylin mRNA transcripts (unpublished data) and protein[13,75] are expressed early in the latter half of gestation starting at embryo day 11/12 in the rat pancreatic diverticulum where amylin is expressed by P-cells[76]. These observations suggest that any function associated with the early fetal expression of this peptide is likely to involve a role for amylin as a growth factor.

### Osteoblasts and Osteoclast Differentiation

Research into the potential effects of amylin on bone density followed the observation that osteopenia was common in diabetic subjects[77,78,79,80]. Indeed, this potential effect of amylin was among one of the earliest physiological effects described following its discovery. It was demonstrated subsequently that amylin acted as a growth factor in bone for the proliferation of osteoblasts[81,82] and more recently in osteoclast differentiation[83].

### Renal Cortex

Amylin is also a potent proliferation factor both during the development of proximal tubules[18] and in the proliferation of adult epithelial cells[53]. The relevance of this action of amylin as a growth factor *in vivo* is further emphasized by the finding that amylin is expressed in the proximal tubules of the developing kidney. This occurs prior to vascularization of the rat kidney with peak levels of amylin mRNA at postnatal day 5 (PN5), some 200-fold higher than found in adult kidney (Fig. 1). This event is tightly regulated, a characteristic of the precise events that regulate growth factors during organogenesis. The identification of the factors that control this tight regulation of amylin expression may prove to be important later in pathogenic processes

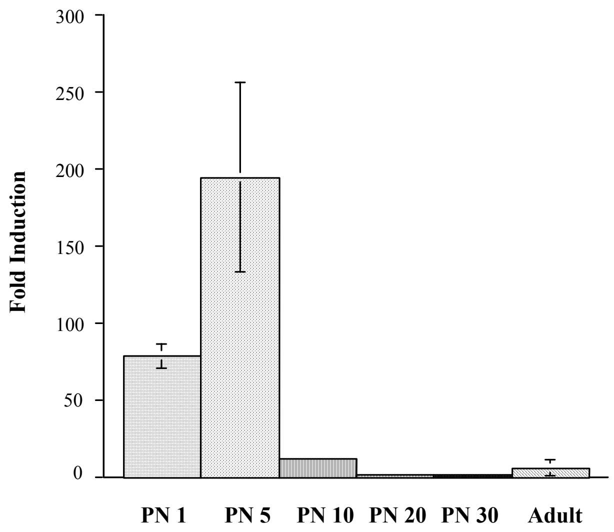

**FIGURE 1**. Real time PCR determinations[17] on developing postnatal (PN day 1–30) rat kidneys to quantify levels of amylin mRNA relative to adult kidney.

such as the development of renal complications associated with diabetes and renal regeneration following other insults.

The analysis of renal development in the amylin gene-deletion (AGD) mouse model[64,84,85] has provided further evidence for the role of amylin as a renal growth factor. First, the profile of levels of amylin mRNA found in the wildtype (WT) mouse kidney (unpublished) is similar to data shown in Fig. 1. By contrast, in AGD mice, amylin mRNA biosynthesis is absent (by real time PCR) indicating that this renal transcript is normally encoded by a single amylin gene. This is corroborated by our finding that the full cDNA sequence of amylin mRNA from rat kidney, as determined using Rapid Amplification of cDNA Ends (RACE), corresponds to the sequence of the mRNA transcript expressed in the islets.

Second, this has detectable consequences as seen in the enlargement of inter-tubular spaces (proximal tubular dropout or a deficiency in the mass of convoluted tubules) in the AGD vs. WT developing kidney and the extension of the proximal epithelial lining into Bowmans capsule which is significantly reduced in adult AGD mice[86].

The physiological consequences of this anatomical abnormality are as yet unclear but it should be noted that these AGD mice clear glucose at a faster rate compared to WT[84,87], which may be related to renal functions in these mice.

## Pancreatic Islets

It has been known for some time that amyloid can induce β-cell death[88]. However a new aspect of β-cell biology has recently been described, which may have significance for the maintenance of β-cell mass. Monomeric amylin has been found to be a growth factor for isolated fetal β-cells[89]. This follows previous descriptions of its effect on cells involved in bone metabolism[81,83,90] and primary cultures of renal proximal tubular epithelial cells[18,53]. Furthermore, amylin may potentiate the effects of other growth factors in inducing proliferation of adult β-cells (E. Karlsson, personal communication).

In this context, we have analyzed the pancreata of adult WT ($n = 3$) vs. AGD ($n = 3$) mice and shown that islet β-cell mass was greatly reduced in the latter. The analyses and volume determinations of pancreata and islets were derived from three-dimensional reconstructions aided by computer imaging techniques using serial sections stained to detect insulin (Fig. 2: upper panels, WT; lower panels, AGD).

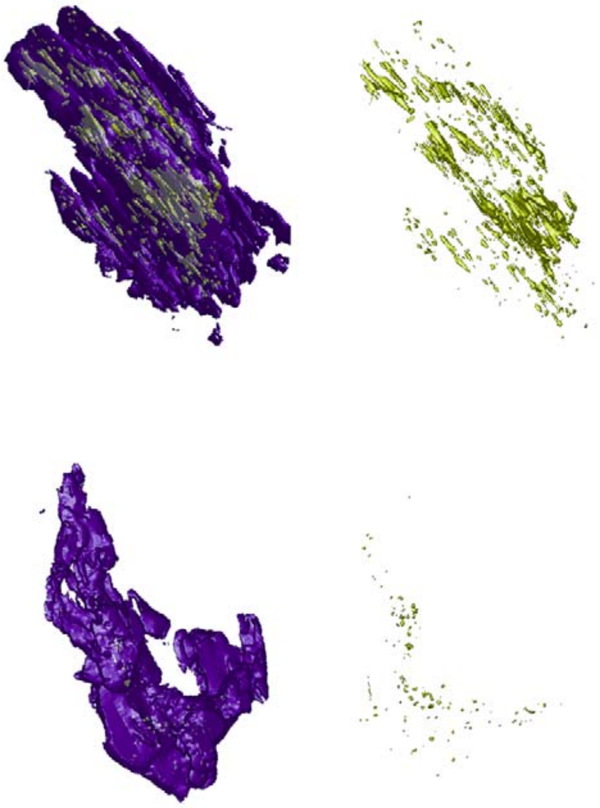

**FIGURE 2**. Representations of three-dimensional reconstructions of total pancreatic tissue (left side) and islet tissue (right side) from WT (top row) and AGD (bottom row) adult mice (see text for results of quantification).

From these data, the total volume of each pancreas and the internal islet volume were calculated (the ratio of total pancreatic volume to islet volume; WT, 51.7; AGD, 2024). The total islet volume of the AGD pancreas was found to be about 1/40$^{th}$ of the WT. There is residual islet mass found in the AGD mice that suggests other factors may act synergistically with amylin, or alternatively may play a role at a different stage in β-cell maturation.

We consider that the reduction in β-cell mass in AGD mice renders them more susceptible to the effects of toxins. Indeed, alloxan induced in these mice more severe diabetes[85]. Decreased β-cell mass was also found in transgenic, diabetic mice expressing human amylin[21,91] presumably due to the toxic effects of amyloid deposits and perhaps combined with a possible reduction in the activity of amylin as a growth factor due to amyloidogenesis.

Furthermore, in pancreatic islets, monomeric amylin may play a role in islet enlargement, an important concept linked to the progression towards overt diabetes. We (unpublished) and others[92] have noted enlarged islets in the fat but not thin Zucker rats, which ultimately became fibrotic. We propose that uncontrolled oversecretion of monomeric amylin in the local environment[93] promotes islet enlargement in this model and results in a prolonged local inflammatory response, which eventually leads to islet fibrosis (unpublished data). These rats have hyperamylinemia that may arise from a number of factors including elevated secretion.

Indeed the genetic defect in these rats is an altered leptin receptor and leptin has been shown to suppress β-cell secretion.

Research aimed at the resolution of the role of amylin as a β-cell specific growth factor is a recent and important aspect of amylin biology. The reduction in the secretion of human amylin in the progression to overt diabetes may have consequences for the maintenance of β-cell mass. There is a 20–50% drop in β-cell mass in NIDDM pancreases as measured in autopsy tissues[21]. Therefore, it is postulated that treatment of subjects with or at risk of diabetes, with a monomeric amylin agonist may result in the preservation or even an increase in β-cell mass.

## BLOOD PRESSURE AND AMYLIN

Amylin at physiological doses has been shown to stimulate renin activity in both rats[54] and man[94], and a modest pressor effect also has been noted[95,96]. This effect may be mediated by insulin-induced changes in renin release[97]. Furthermore, in rat models of renal hypertension, activation of renal amylin–binding sites could be correlated with blood pressure[4,5,56,98]. These high-affinity sites are located on proximal tubules[53,54,56]. More recently, amylin has been reported to induce relaxation of aortic rings[99], inhibit CCK-induced contraction of smooth muscle cells[100], and modulate pulmonary vascular tone[101]. Both the vasodilator effects of amylin on the mesenteric vasculature[102] and similar effects on coronary and basilar arteries are mediated by CGRP type 1 receptors[103,104]. However, in whole animal experiments with AGD mice, no altered blood flow rates in the whole pancreas, pancreatic islets, duodenum, ileum or colon were observed[105].

## AMYLIN HIGH-AFFINITY BINDING SITES AND PUTATIVE RECEPTORS

### Calcitonin Receptors (CTR)/Receptor Activity Modifying Proteins (RAMP) Hypothesis

It has been proposed that the affinity of the CTRs and CTR-like receptor (CRLR) may be modified by interactions with RAMPs[106,107]. Thus the pharmacological binding characteristics of the CTRs may be altered by coexpression of RAMPs 1, 2, or 3 as demonstrated with transfected cell lines. For instance, RAMPs 1 and 3 may combine with human CTR and result in a receptor with greater affinity for rat amylin[108,109].

### High-Affinity [$^{125}$I]-Amylin Binding Sites in Kidney and CNS

A number of anti-CTR antibodies have now been developed and characterized[17,110,111]. There is some discrepancy between the distribution of CTR ([17], Fig. 3A), which appears to be colocalized with RAMP-3 expression (Fig. 3B) in the distal tubules, collecting ducts, and ascending limbs of the loops of Henle, and high-affinity amylin binding sites[54] that have been found on the proximal tubules[53]. These data suggest that the renal amylin receptor is yet to be identified and characterized, and that it is most probably not related to CTR.

Similar studies[111] need to be performed in the CNS where high-affinity amylin binding sites have also been mapped[71,72]. Studies on the distribution of RAMP mRNA transcripts in the CNS have recently been reported[100,101]. An important issue is that further mapping studies should address concerns about the correlation of the locations of amylin binding sites with the

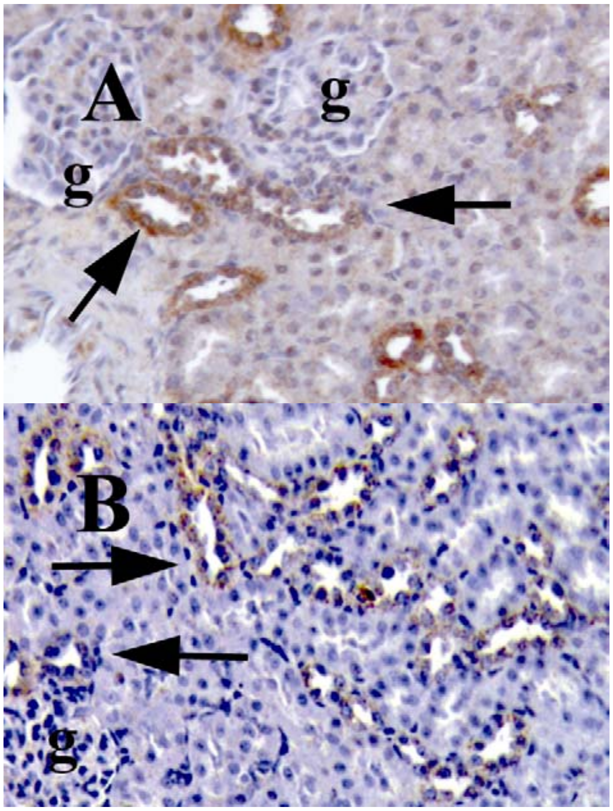

**FIGURE 3**. Immunohistochemical staining of rat PN30 kidney using (A) a monoclonal anti-CTR antibody[17] and (B) an anti-RAMP 3 antibody (Alpha Diagnostic Int).

expression of CTR isoforms and RAMP accessory proteins in the CNS. These important studies will aid in the identification of physiologically relevant amylin binding sites and receptor composition.

## CONCLUSIONS

In this review we have concentrated on the recent research publications that address issues related to the biology of amylin. Of central importance to the etiology of diabetes, are the factors that contribute to the uncoupling of the regulation of β-cell–secreted products and atrophy of the β-cells themselves. The latter is dependent on the nature of amyloid deposits, the sensitivity of the β-cells, and the potency of amylin as a growth factor in this pathophysiological state. A study on the efficacy of an amylin nonpeptide agonist in this context would be useful to determine if the mass of β-cells can be conserved in a functional form.

Furthermore, in diabetic states little is known about expression from secondary sites of amylin biosynthesis discussed above and any significance that this might have for the etiology of diabetes and associated complications.

It is also debated whether amylin and/or prolonged activation of its receptors are central players in the maintenance of blood pressure and development of hypertension, respectively.

The question of the nature of amylin binding sites in the kidney, CNS, and elsewhere has yet to be resolved and the relation of these high-affinity binding sites to the CTR/RAMP hypothesis, which was derived from data generated from coexpression in cell lines.

Amylin peptide antagonists have been used in a number of research projects to discern the functions of amylin in the periphery. Nevertheless, the final identification and verification of the physiological receptors for amylin should lead in the future to the characterization of amylin nonpeptide analogues, and is viewed as important for the future development of this field. Most importantly, these compounds may be useful to slow down the onset of diabetes and the treatment of related disorders such as diabetic nephropathy and hypertension.
